# Sonoporation efficacy on SiHa cells in vitro at raised bath temperatures—experimental validation of a prototype sonoporation device

**DOI:** 10.1186/s40349-015-0040-9

**Published:** 2015-11-06

**Authors:** Jonathan Kivinen, Melissa Togtema, Gregor Mulzer, Joshua Choi, Ingeborg Zehbe, Laura Curiel, Samuel Pichardo

**Affiliations:** Department of Electrical Engineering, Lakehead University, 955 Oliver Road, Thunder Bay, Ontario, P7B 5E1 Canada; Image-Guided Interventions, Thunder Bay Regional Research Institute, 980 Oliver Road, Thunder Bay, Ontario, P7B 6V4 Canada; Department of Biology, Lakehead University, 955 Oliver Road, Thunder Bay, Ontario, P7B 5E1 Canada; Probe Development and Biomarker Exploration, Thunder Bay Regional Research Institute, 980 Oliver Road, Thunder Bay, Ontario, P7B 6V4 Canada

**Keywords:** Sonoporation, Drug delivery, Ultrasound, Transducer array, In vitro, Temperature, HPV, SiHa

## Abstract

**Background:**

A device was devised which aimed to reduce the time and expertise required to perform sonoporation on adherent cell cultures. This prototype device was used to examine the superficial effect of bath temperature on sonoporation efficacy.

**Methods:**

The prototype device consisted of six ultrasound transducers affixed beneath an Opticell stage. Six transducers with nominal diameters of 20 mm were constructed and the acoustic field of each was characterized using hydrophone scanning. A near field treatment plane was chosen for each transducer to minimize field heterogeneity in the near field.

Cervical cancer-derived SiHa cells were exposed to nine different treatments in the presence of plasmid DNA-expressing green fluorescent protein (GFP). Ultrasound treatment with Definity ultrasound contrast agent (US+UCA) present, ultrasound treatment without contrast agent present (US), and a sham ultrasound treatment in the presence of ultrasound contrast agent (CA) were each performed at bath temperatures of 37, 39.5, and 42 °C. Each treatment was performed in biological triplicate. GFP expression and PARP expression following treatment were measured using fluorescent microscopy and digital image processing. Cell detachment was measured using phase contrast microscopy before and after treatment.

**Results:**

Mean (± s.d.) transfection rates for the US+UCA treatment were 5.4(±0.92), 5.8(±1.3), and 5.3(±1.1) % at 37, 39.5, and 42 °C, respectively. GFP expression and cell detachment were both significantly affected by the presence of ultrasound contrast agent (*p* < 0.001, *p* < 0.001). Neither GFP expression, PARP expression, or detachment differed significantly between bath temperatures.

**Conclusions:**

Bath temperature did not impact the efficacy of sonoporation treatment on SiHa cells in vitro. The prototype device was found to be suitable for performing sonoporation on adherent cell cultures and will reduce the time and expertise required for conducting sonoporation experiments on adherent cell cultures in the future.

## Background

Sonoporation is a process in which ultrasound exposure can induce a transient increase in the permeability of cell membranes. This process has been shown to be highly dependent on the presence of cavitation nuclei [1–3]. In practice, clinically approved ultrasound contrast agents, comprised of microscale inert gas bubbles (“microbubbles”) often having lipid, polymer, or protein shells, are added to cell media to serve as cavitation nuclei during ultrasound exposure.

Microbubble cavitation is known to induce a number of biophysical effects. At low acoustic intensities, stable cavitation has been shown to induce shear stress on the cell membrane by the generation of rapid flow in the surrounding medium, referred to as “microstreaming” [4–6]. In [4], the authors captured high-speed images of oscillating microbubbles pushing and pulling on the cell membrane when in close proximity, an interaction which correlated with increased uptake of propidium iodide. At higher acoustic pressures, inertial cavitation can occur, resulting in the formation of shock waves as well as “microjets” in the presence of a relatively rigid cell membrane [7, 8]. The stresses on the cell membrane due to cavitation can result in physical damage to the cell membrane allowing for the passive diffusion of agents across [4, 9].

In addition to physical disruption of the cell membrane, cavitation has been shown to provoke other biological effects. For example, there is evidence that endocytosis plays a role in the uptake of agents during sonoporation. In [9], the authors observed that following ultrasound-mediated uptake of dextrans into primary endothelial cells, the smaller dextrans used (4.4 and 70 kDa) were homogeneously distributed in the cytosol whereas the larger dextrans used (155 and 500 kDa) were heterogeneously distributed and encapsulated by endocytotic vesicles. The authors then examined the effect of a number of endocytosis-inhibiting treatments and observed significant decreases in uptake of dextrans from sonoporation. Though decreases were seen in the uptake of smaller dextrans, the decrease in uptake of the larger dextrans was much more pronounced.

Sonoporation is of particular interest to those in the field of cancer research as it promises to provide a non-viral means for targeted drug delivery and gene therapy. High-risk human papillomavirus (HPV) infection is strongly associated with the development of cervical cancer, the second most common cancer affecting women worldwide [10]. Currently, standard treatment of cervical cancer includes radiation therapy and surgery, each having their own undesirable side effects. Ultrasound-mediated delivery of therapeutic macromolecules which target the oncoproteins encoded by the HPV DNA (e.g., plasmid DNA [2, 11] and small interfering RNA [12, 13]) may provide a minimally invasive alternative to current treatment options. In vitro studies on cell cultures are an essential part of discovery and testing of therapeutic agents which can be delivered using this process.

There have been a few experimental setups described in the literature for performing sonoporation in vitro on adherent cell cultures. The cells are typically adhered to either a well plate, Petri dish [13–17], or the inner membrane of an Opticell cell culture chamber (Thermo Scientific Nunc, Waltham, MA, USA) [2, 12, 18–23]. The use of well plates and Petri dishes located at the surface of the ultrasound bath is problematic since the acoustic conditions at the media-air interface may not necessarily be known due to the possibility of reflection and standing wave formation [14]. In [13], the authors use an elaborate setup to eliminate the media-air interface at the well plate which involves placing a second water bath on the far side of the well plate with respect to the acoustic source.

The Opticell cell culture chambers consist of two thin, parallel, gas-permeable, cell culture-treated polystyrene membranes. These chambers allow for adherent cell culture with sterile media and appropriate oxygen and carbon dioxide levels, while facilitating ultrasound exposure through a minimally reflective surface [2]. Their ability to be fully submerged in the ultrasound bath allows for an acoustic absorber to be placed in the acoustic far field, reducing the effects of reflection and standing wave formation.

Due to the growth area of an Opticell (50 cm^2^), multiple acoustic exposures are often performed across the cell culture area with a single transducer and the aid of a positioning system [12, 15, 18, 19, 21]. In order to ensure consistent acoustic exposure, the cell culture, positioning system, and transducer must be well aligned. Our group has used an experimental setup consisting a three-axis, computer-controlled micropositioning system in a large (approximately 200 L) water tank (UMS2; Precision Acoustics, Dorsetshire, UK) for sonoporation studies [12, 18, 24]. Aligning the cell culture and configuring the experiment software was a long and involved process, requiring experience with software programming, electronics, and acoustics. For instance, the positions of three known points on the cell culture stage needed to be localized by pulse-echo and scanning techniques in order to determine its orientation. Once the cell culture stage was aligned and the software was configured, each exposure was performed sequentially, requiring time between each target for the transducer to travel and rest. The time required for the large water bath to be filled, degassed, heated to a biologically relevant temperature, and emptied also consumed a large portion of the experiment day.

Here, a device for sonoporation of adherent cell cultures is presented. This device aims to reduce the time and expertise required for experiment setup with respect to our previous setup. The device was constructed and characterized before being experimentally validated. Additionally, as part of a preliminary study of the effects of temperature on sonoporation, this device underwent testing at bath temperatures 37, 39.5, and 42 °C representing a temperature range from biological temperature to hyperthermia. The rationale of studying the effect of temperature was to foresee a potential synergistic effect of mild thermal effects in the transfection efficiency, which can help to better design translational applications of sonoporation.

## Methods

### Sonoporation device

**Fig. 1:**
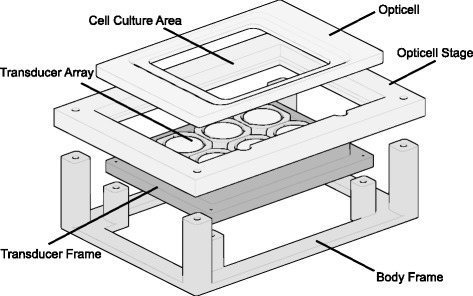
A computer rendering (exploded) of the device illustrating the different parts. A computer rendering (exploded) of the device illustrating the different parts. The transducer array body and cell culture stage are affixed to the body frame by a set of screws. The Opticell is press-fit into the Opticell stage before treatment by the operator and can easily be removed by hand

A sonoporation device was devised using an array of ultrasound transducers and a cell culture stage designed for use with the Opticell cell culture system (Fig. 1). The transducer array consisted of six air-backed transducers, each having a square (26 × 26 mm) housing, 25 mm tall. The transducer housings were fabricated from ABS (acrylonitrile-butadiene-styrene) plastic (Makerbot Industries, New York, NY, USA) using a desktop 3D printer (Makerbot Replicator; Makerbot Industries, New York, NY, USA). Each transducer featured a flat, circular piezoelectric crystal with a nominal diameter of 20 mm (DL-47; Del Piezo Specialties, LLC, West Palm Beach, FL, USA). The device was designed such that the transducer array was affixed beneath the Opticell stage in order to maintain the distance between the transducers and the target cells between experiments, greatly minimizing the time and training requirements for setting up the acoustic conditions. The cell culture stage, transducer frame, and body frame were also fabricated with ABS via 3D printing. Each transducer was electrically matched to 50 *Ω* at its operating frequency (1 MHz) using an external low-pass L-type matching circuit with the aid of a network analyzer (HP 8127ES; Hewlett Packard, Palo Alto, CA, USA).

**Fig. 2:**
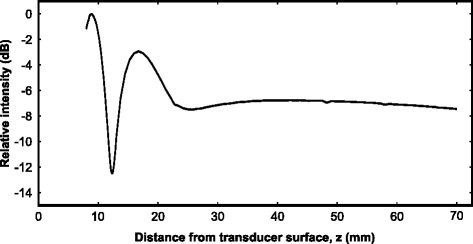
Acoustic intensity along the acoustic axis of a select transducer. The acoustic intensity measured down the center of the acoustic axis (*z*) from *z* = 8 mm to *z* = 70 mm in steps of *Δ* *z* = 0.1 mm for 8–13 mm and *Δ* *z* = 0.7 mm for 13–70 mm

**Fig. 3:**
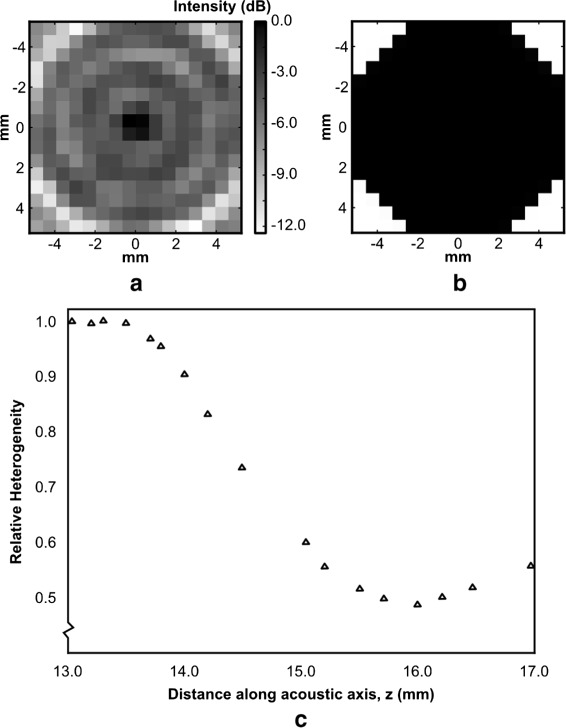
Treatment profile heterogeneity. **a** The measured acoustic profile of a 10.5 × 10.5 mm plane perpendicular to the beam path for one of the transducers where *Δ*x = *Δ*y = 0.7 mm < 0.5 *λ*. **b** The mask used to select a circular region for calculating homogeneity in **a**, where *black* represents the selected area. **c** The measured “heterogeneity” of each slice at different points along the acoustic axis from 13 to 17 mm for one of the transducers, showing a local minimum at *z* = 16 mm. This point was chosen as the optimal near field treatment distance for this transducer

The device was chosen to operate in the near field of the transducers in order to minimize its size. The acoustic intensity profile of each transducer was obtained using the planar scanning method using a calibrated needle hydrophone (0.2 mm, SN1426; Precision Acoustics, Dorsetshire, UK) and a motorized micropositioning system (UMS2; Precision Acoustics, Dorsetshire, UK) at low acoustic pressures. The acoustic intensity down the center of the beam path was obtained from 8 to 70 mm (Fig. 2). A range of 10–20 mm was explored for a treatment plane as a trade-off between peak intensity and treatment distance (homogeneity). Slices perpendicular to the beam path (10.5 × 10.5 mm, *Δ*= 0.7 mm) were obtained on this range and characterized for field homogeneity (Fig. 3a). Homogeneity was quantified as the standard deviation of the pressure over the circular area encompassed by the planar scan, centered with the acoustic axis (Fig. 3b). The distance at which the minimum standard deviation occurred was chosen as the optimal treatment distance for the transducer (Fig. 3c). This distance was determined for each transducer. Finally, on each optimal plane, a point of approximately 90 % relative intensity was chosen in order to determine the electrical power required to produce the desired peak-negative pressure. The input electrical voltage for each transducer was adjusted to achieve the acoustic power required for the ultrasound exposures.

**Fig. 4:**
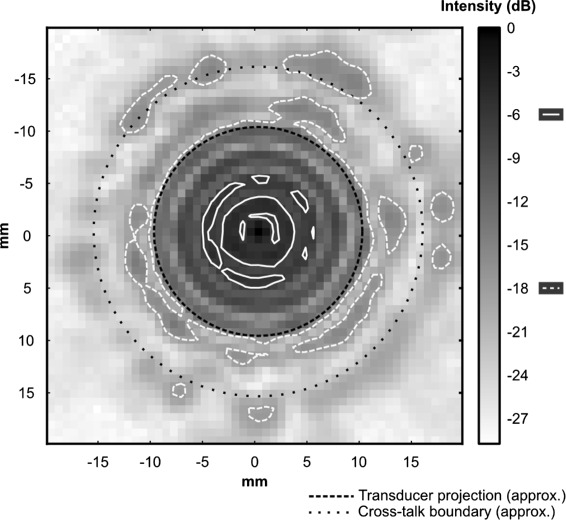
Device cross-talk in array. A wide scan (40 × 40 mm, *Δ* = 0.7 mm) of one of the transducers at its treatment distance taken to examine the field intensity near surrounding transducers in the array. Denoted in *white* are the −6 (*solid*) and −18 dB (*dashed*) contours and in *black* are an approximation of the orthographic projection of the source transducer (*dashed*) and the approximate distance to the edge of a neighbouring transducer (*dotted*). The −18 dB contour shows that the intensity at the edge of neighbouring transducers is low (<−16 dB)

One of the six transducers was measured over a wider plane (40 × 40 mm, *Δ*= 0.7 mm) to examine the amount of cross-talk from one transducer to the treatment area of another (Fig. 4) and was found to be sufficiently low (<−16 dB).

### Experimental groups

Experiments were divided into nine groups consisting of three different treatments and three different temperatures. Cells either underwent a sonoporation treatment (US+UCA) which exposed the cells to high-intensity ultrasound with UCA present, an ultrasound treatment (US) which exposed the cells to high-intensity ultrasound without UCA present, or a sham ultrasound treatment (UCA) which used zero acoustic power (amplifier turned off) with UCA present. Each of these three treatments was performed at 37, 39.5, and 42 °C with plasmid DNA present. These three temperatures represent a temperature range from biological temperature to hyperthermia. All nine experimental groups were repeated three times.

### Ultrasound exposure

The ultrasound generation parameters used in the experimental validation were adopted from previous studies by our group [12, 18] in order to compare its relative performance with our previous system. These parameters were originally chosen based on preliminary work by our own group [24]. Pulsed ultrasound at 1 MHz delivered at a peak-negative pressure of 0.7 MPa to the cell culture in bursts of 30 cycles every 625 *μ*s (4.8 % duty cycle; 1.6 kHz pulse repetition frequency) for a total time of 30 s.

The ultrasound signal for performing both characterization and sonoporation was generated using a waveform generator (33522; Agilent Technologies Canada Inc., Mississauga, Ontario, Canada) and amplified using a linear, radio-frequency power amplifier (A150; E&I, Rochester, NY, USA). The power during sonication was monitored using an in-line directional coupler (C5685-10; Werlatone Inc., Patterson, NY, USA) and power meter (2 × N8482H sensors and N1914A meter; Agilent Technologies Canada Inc.). This setup allowed for the excitation of one transducer at a time during experiments. Hence, each treatment area of the cell culture chamber was treated sequentially in random order. An additional period of 30 s after each exposure was added to allow the operator to switch to the next transducer in the array.

### Cell culture

Cervical cancer-derived SiHa cells (ATCC HTB-35, Manassas, VA, USA) were used in this study. Such cells contain 1–2 genome copies of HPV type 16 per cell [25]. The cells were maintained in 75-cm^2^ flasks containing Dulbecco’s Modified Eagle Medium (DMEM; Sigma-Aldrich, Oakville, Ontario, Canada) supplemented with 10 % heat-inactivated fetal bovine serum (FBS; Hyclone Laboratories Inc., Logan, UT, USA), 100 U of penicillin, 100 *μ*g of streptomycin, and 0.25 *μ*g amphotericin B per mL (antibiotic/antimycotic; Gibco, Grand Island, NY, USA) at 37 °C and 5 % CO_2_. The cells were passaged to maintain 70–80 % confluency. Twenty-four hours before treatment, the cells were seeded (0.6 × 10^6^) into an Opticell chamber to allow the cells to adhere to the inside membrane.

Immediately following treatment, the Opticell was removed from the ultrasound bath, wiped with 70 % ethanol, and returned to the incubator where the cells were incubated at 37 °C. After 2 h, 1.1 mL of serum-free media was removed from the Opticell and the remaining media was supplemented with 1 mL of FBS and 100 *μ*L of antibiotic/antimycotic, returning the cells to their original media composition. The cells were then incubated for an additional 24 h to allow for GFP expression by the cells.

### Plasmid DNA

Plasmid DNA (pDNA) expressing green fluorescent protein (GFP) was used to quantify successful transfection. To produce copies of the plasmid, chemically competent NEB 5- *α*F’I^q^*Escherichia coli* bacteria (New England Bio Labs Inc., Ipswich, MA, USA) were transformed with 6.3-kb Omicslink pReceiver-M03 plasmid containing the GFP complementary DNA (Genecopia Inc., Rockville, MD, USA). Plasmid DNA was extracted and purified with EndoFree Plasmid Maxi Purification kits (Qiagen Inc., Toronto, Ontario, Canada) to minimize bacterial endotoxin levels. Prior to treatment, the cells were washed with serum- and antibiotic-free DMEM and incubated with 250 *μ*g of plasmid DNA in 10 mL of serum and antibiotic-free DMEM for 15 min at 37 °C and 5 % CO_2_ to allow the plasmid DNA to diffuse within the media.

### Ultrasound contrast agent

Definity ultrasound contrast agent (Lantheus Medical Imaging, North Billerica, MA, USA), consisting of bubbles of a perflutren gas core and lipid shell, was used to introduce cavitation nuclei during the sonoporation process. The contrast agent was activated according to the manufacturer’s recommended procedures (Vialmix, Lantheus Medical Imaging, North Billerica, MA, USA). For those experimental groups which included contrast agent, 33 *μ*L of activated contrast agent was added to the 10-mL media in the Opticell chamber 1 min before transferring to the water bath, giving a final volume concentration of 0.33 %. Nominally, activated Definity contains 1.2 × 10^10^ microbubbles per milliliter corresponding to a microbubble-cell ratio of 660:1. During treatment, the Opticell was placed horizontally in the cell culture stage with the cells on the upper-most membrane, allowing for the microbubbles to rise and rest against the cells during insonation.

### Bath conditioning and heat treatment

**Fig. 5:**
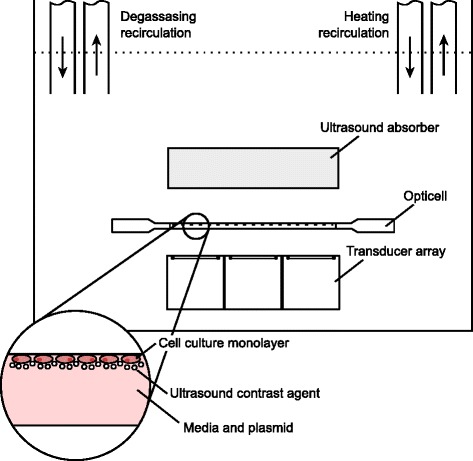
Sonoporation experimental setup schematic. Sonoporation setup featuring a transducer array below an Opticell chamber. Beyond the cell culture is an ultrasound-absorbent material (Aptiflex; Precision Acoustics, Dorsetshire, UK) intended to reduce reflection and standing wave formation

Experiments were conducted in a bath of deionized, degassed water (Fig. 5). The bath water was initially circulated through a degassing system until the detectable level of dissolved oxygen was <1.0 ppm (407510A; Extech Instruments Corporation, Nashua, NH, USA). The water was also circulated through an in-line water heater (Model 210; PolyScience, Niles, IL, USA) to raise it to the desired temperature. The heater continued to circulate water throughout the duration of the experiment in order to maintain the temperature of the bath. The compact dimensions of the device allowed for experiments to be performed in a small water tank (a 26 L tote) reducing the time consumed by filling and emptying the tank as well as degassing and heating the water bath.

The cells and medium in the Opticell chamber were given 1 min to equalize with the temperature of the surrounding bath before starting the ultrasound exposure. This value was determined experimentally by measuring the time that water in an Opticell chamber took to rise from the temperature of the incubator (37 °C) to the maximum bath temperature tested (42 °C). Due to the large ratio between the surface area and the volume of the Opticell, the average time for this rise to occur was approximately 40 s, which was rounded up to 1 min to ensure the temperature had stabilized.

Considering the initial temperature equalization time and subsequent treatment time, each Opticell was submerged in the bath for a total of 7 min.

### Quantification of transfection and viability

Transfection efficiency and viability were quantified using microscopy and image processing. Cell imaging was performed using a Zeiss Axiovert 200 inverted microscope (Carl Zeiss Canada Ltd., North York, Ontario, Canada) and an LD A-Plan 10 ×/0.25 Ph1 objective for a total magnification of 100 ×. A 12-bit CCD camera (Q Imaging, Surrey, British Columbia, Canada) was used to capture the microscope images to digital format for processing.

Cell loss (detachment) was evaluated by imaging the cell cultures in three random spots per exposure area (18 total per replicate) 15 min before and 2 h after treatment with phase contrast imaging. Each cell was manually identified by applying an identifying marker (a dot) over the digital phase contrast images and subsequently creating a new image containing only the dots on a blank background. These marked images were then used to automatically obtain cell counts per field of view using CellProfiler software (2.0.0) [26]. The cell counts per field of view were averaged per replicate for analysis. The cell loss for each replicate was evaluated as the relative change in the average cell count 15 min before and 2 h after treatment where a negative relative change represented a drop in cell count. It was assumed that any cells that detached were non-viable. The viability of the remaining cells was determined by visualizing cleaved poly (ADP-ribose) polymerase (PARP), an early apoptotic indicator, 24 h after treatment.

Twenty-four hours following treatment, the Opticell membrane with the adhered cells was removed and the cells were fixed with a 4 % solution of paraformaldehyde (PFA) in phosphate-buffered saline (PBS). The fixed cells were permeabilized with a 0.1 % solution of Triton-X in PBS for 5 min and rinsed with PBS. The cells were then blocked with a 1 % solution of bovine serum albumin (BSA) in PBS for 10 min at room temperature. Since the fluorescent signal produced by the GFP was quenched by the PFA fixing, a goat polyclonal anti-GFP antibody (ab5450; Abcam Inc., Toronto, Ontario, Canada) was applied at 1:1000 in 1 % BSA/PBS to bind to the GFP protein produced by transfected plasmid. A green fluorescent AlexaFluor 488 donkey anti-goat secondary antibody (LifeTechnologies Inc., Burlington, Ontario, Canada) was applied at 1:400 in 1 % BSA/PBS to visualize the antibodies bound to the GFP. A monoclonal mouse anti-cleaved PARP antibody (ab1103315; Abcam Inc., Toronto, Ontario, Canada) was applied at 1:760 in 1 % BSA/PBS and visualized with a secondary red fluorescent Alexa Fluor 594 donkey anti-mouse antibody (LifeTechnologies Inc., Burlington, Ontario, Canada) applied at 1:800 in 1 % BSA/PBS. The cells were counter-stained with 4’,6-diamidino-2-phenylindole (DAPI) to identify the nuclei of the cells before being mounted onto slides for imaging. DAPI was already incorporated directly into the medium use to mount the cells on the Opticell membranes.

**Fig. 6:**
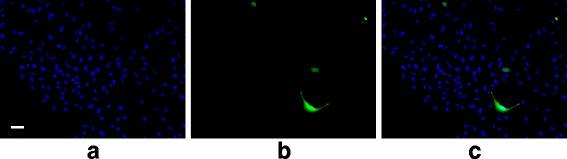
Fluorescent microscopy example showing transfected cells. An example field of view from the US+UCA group showing **a** DAPI (nuclei), **b** GFP antibody (transfected), and **c** DAPI and GFP antibody fluorescence overlaid. *White line* denotes 100 *μ*m

**Fig. 7:**
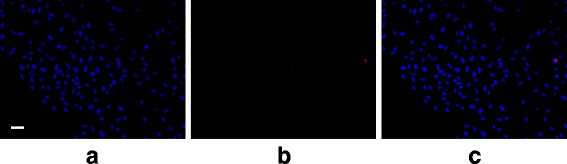
Fluorescent microscopy example showing apoptotic cells. An example field of view from the US+UCA group showing **a** DAPI (nuclei), **b** cleaved PARP antibody (apoptosis), and **c** DAPI and cleaved PARP antibody fluorescence overlaid. *White line* denotes 100 *μ*m

The stained cells were imaged in five random spots per treatment area using green, blue, and red fluorescence filters. One of the six treatment areas was used as a staining control, limiting the total available fields of view for analysis to 25 per replicate. Each field of view was captured for each of the three stains (Figs. 6 and 7).

Object segmentation was performed on the fluorescent microscopy images using CellProfiler (2.0.0) [26]. Two types of “objects” (independent areas of the image) were extracted in each field of view: (1) the nuclei objects, extracted from the blue DAPI images; and (2) cell boundary objects, extracted using a combination of the blue DAPI and green GFP secondary antibody images. These objects were extracted using an Ostu-global segmentation method with minimization of weighted variance. The extracted objects represented areas of the image occupied by individual nuclei (nuclei objects) or cells (cell boundary objects). Consequently, the background of the image was identifiable, based on the area not occupied by any object.

For an object to be considered positive for either transfection (green) or apoptosis (red), the image intensity inside the object should be significantly higher than the image intensity of the background. Thus, the following criterion was used to detect positive objects:

(1) $$ \bar{I}_{\text{obj}} \ge \bar{I}_{\text{bg}} + n\sigma_{\text{bg}}   $$

where $\bar {I}_{\text {obj}}$ is the mean image intensity within an object, $\bar {I}_{\text {bg}}$ is the mean intensity of the background, *σ*_bg_ is the standard deviation of the background intensity, and *n* is the number of standard deviations that the mean object intensity must be from the mean background intensity for the object to be considered positive. In [12], a value of *n*=2 was used with this method. This threshold was increased to *n*=3 in this work. A cell was considered transfected if it met this criterion using the green GFP secondary antibody images. Cell boundary objects were used in transfection detection since the GFP secondary antibody signal would be observed in both the cytoplasm and nucleus of each positive cell. This same criterion was used for detecting cells positive for apoptosis using the intensity of the red cleaved PARP secondary antibody images. Since the cleaved PARP secondary antibody signal would be observed in the nucleus of each positive cell, the nuclei objects were used. The number of objects positive for transfection and apoptosis were counted relative to the number of cells in the image. The fraction of positive objects within each field of view was averaged per replicate for statistical analysis.

### Statistical analysis

Statistical analysis was performed using R (2.15.2) [27]. Observations were tested for normality using the Shapiro-Wilk test and homogeneity of variance using Bartlett’s test. A two-way ANOVA was used to test the effects on parametric data. A post hoc Tukey’s HSD (honestly significant differences) test was performed on significant effects. For non-parametric data, the Kruskal-Wallis rank sum test was used to test effects and a post hoc pair-wise Wilcoxon rank sum test was performed for significant effects. The significance level (*α*) was made 0.05 a priori.

## Results

### Transfection efficiency

**Fig. 8:**
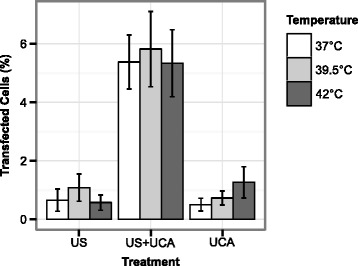
Rates of transfection for each treatment at each temperature. Transfection rates for each treatment and temperature tested. Post hoc analysis showed that the US+UCA treatment group had significantly higher percentage of transfected cells over the other two treatment groups independent of temperature (*p* < 0.001 for both). There was no significant effect observed in transfection rates with temperature or treatment-temperature mixed effects. *Error bars* represent ± s.d. (*n* = 3 per group)

Transfection rates for each experimental group are shown in Fig. 8. For temperatures of 37, 39.5, and 42 °C, the average (±s.d.) percentages of transfected cells for the sonoporation treatment (US+UCA) were 5.4(±0.92), 5.8(± 1.3), and 5.3(±1.1) %, respectively; the percentages of transfected cells for the ultrasound treatment (US) were 0.66(±0.38), 1.1(±0.46), and 0.57(±0.26) %, respectively; and the percentages of transfected cells for the sham treatment (UCA) were 0.50(±0.22), 0.73(±0.24), and 1.3(±0.53) %, respectively.

Treatment was observed to have a significant effect on transfection efficiency (*p* < 0.001). Post hoc analysis showed that the cells that received the sonoporation treatment (US+UCA) had a significantly higher expression of GFP over the control treatments (*p* < 0.001 for both). Transfection efficiency was not observed to be significantly affected by bath temperature (*p* = 0.564) or treatment-temperature interaction effects (*p* = 0.684).

### Cell viability

**Fig. 9:**
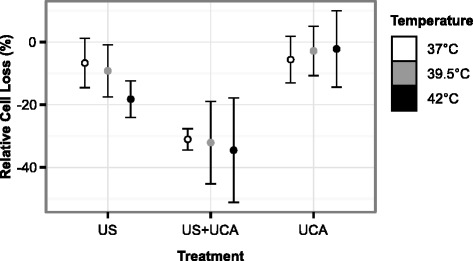
Rates of cell loss for each treatment at each temperature. Relative change in cell count following treatment (+ 2 h) for each treatment and temperature. Post hoc analysis showed US+UCA treatment has significantly higher cell loss over the other two treatments (*p* < 0.001 for both). There was no significant effect observed in transfection rates with temperature or treatment-temperature mixed effects. *Error bars* represent ± s.d. (*n* = 3 per group)

Rates of cell loss following treatment for each experimental group are shown in Fig. 9. For temperatures of 37, 39.5, and 42 °C, the cell losses for the US+UCA treatment group were −31(±3.4), −32(±13), and −34(±17) %, respectively; cell losses for the US treatment group were −6.7(±7.9), −9.1(±8.3), and −18(±5.9) %, respectively; and cell losses for the UCA treatment group were −5.6(±7.4), −2.8(±7.9), and −2.2(±12.2) %, respectively.

Cell loss was not significantly affected by temperature (*p* = 0.661) or treatment-temperature mixed effects (*p* = 0.778). However, cell loss was found to be significantly affected by treatment (*p* < 0.001). Post hoc analysis showed that US+UCA treatment had significantly higher levels of cell loss over the US and UCA treatment groups (*p* = 0.0076 and *p* = 0.0002, respectively).

**Fig. 10:**
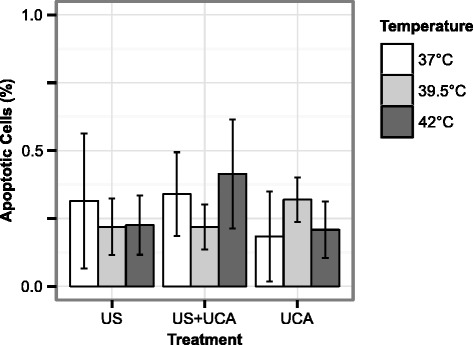
Rates of apoptosis for each treatment at each temperature. The ratio of apoptotic cells for each treatment and temperature. Apoptosis rates were low (<0.75 %) across all treatments and temperatures. No significant effects on apoptosis rates were observed with treatment, temperature, or mixed effects. *Error bars* represent ± s.d. (*n* = 3 per group)

Rates of apoptosis for each experimental group are shown in Fig. 10. For temperatures of 37, 39.5, and 42 °C, the ratios of apoptotic cells for the US+UCA group were 0.34(±0.15), 0.22(±0.08), and 0.41(±0.2) %, respectively; the ratios of apoptotic cells for the US group were 0.31(±0.25), 0.22(±0.10), and 0.23(±0.11) %, respectively; and the ratios of apoptotic cells for the UCA group were 0.18(±0.17), 0.32(± 0.08), and 0.21(±0.10) %, respectively.

The percentage of remaining cells indicating apoptosis 24 h following treatment was low (<0.75 %) and was not significantly affected by treatment (*p* = 0.437), temperature (*p* = 0.896), or treatment-temperature interaction effects (*p* = 0.371).

## Discussion

The sonoporation device presented here was able to induce significantly higher transfection of GFP plasmid DNA over the control groups 24 h following treatment. This device was chosen to operate under the similar excitation parameters as our previous system in order to compare its relative performance. In [18], our previous system produced a transfection efficiency of 6 (±2) % with a cell detachment rate of −47(±32) % using similar excitation parameters, plasmid, and cell line. At 37 °C, the system presented here was able to achieve a transfection rate of 5.4(±0.92) % with a cell detachment rate of −31(±3.4) %. The rates seen here are slightly lower than those seen with our previous system. As transfection has been noted to be dependent on the acoustic pressure [1, 3], a lower rate of transfection may be due to the use of 0.7 MPa here which is lower than the 1 MPa used in [18].

To the best of our knowledge, there are no other groups performing sonoporation on SiHa cells in particular. Furthermore, the acoustic excitation parameters, acoustic conditions, and impermeable agent used vary across the literature, making comparisons between different studies difficult. There are a number of studies on adherent cell cultures which exhibit similar rates of transfection albeit under different acoustic and experimental conditions. For example, in [2], sonoporation using 1 MHz of pulsed ultrasound was examined under a number of different excitation parameters using a near field setup. The authors found that sonoporation at their optimal parameters (1 MHz, 0.25 MPa peak-negative pressure, 4 % duty cycle, 1 kHz PRF, 10 s exposure, 4 % SonoVue UCA concentration) was able to deliver plasmid DNA with an efficiency of ∼4 % to Chinese hamster ovary (CHO) cells with ∼90 % cell viability. A direct comparison to more established transfection methods such as chemical action was not performed for simplicity reasons. Transfection efficiency with chemical methods also depend on multiple factors including cell membrane conditions, nucleic acid/chemical ratio, among others, thus affecting considerably the transfection efficiency in function of the cell line [28]. For example, non-published data from our group on chemical transfection on NIKS cells (which are also adherent, epithelial cells like SiHa, but HPV negative) and a concentration of 0.5 *μ*g/mL plasmid DNA showed transfection efficiencies ranging from 3.4 to 14 %, depending on the experimental replicate, which indicates a strong variability depending on the mentioned conditions.

The presence of UCA during ultrasound exposure not only had a significant effect on transfection but it also appeared to have a significant effect on cell detachment. There have been a number of reports of high levels of cell detachment among the same order as seen here [2, 19, 29]. The level of cell detachment due to ultrasound exposure alone (US group) was on the same order as the sham treatment (UCA group) at 37 °C. Since the US+UCA group had significantly higher cell detachment over US alone and UCA alone, microbubble cavitation may be facilitating cell detachment. In [30], high-pressure shock waves were used to induce cavitation in an adherent cell culture. Using high-speed imaging, the authors found that cell detachment only occurred when cavitation activity was present. The reason for this, they suggested, was that the flow due to the cavitating bubble near the rigid substrate generated a shear force large enough to remove the cells from the substrate. Though the rarefactional pressure of the shock waves in [30] was large enough to induce cavitation without UCA present, a similar effect may have occurred at a lower pressure amplitude here due to the presence of the UCA.

The preliminary study of temperature on sonoporation efficacy resulted in no observable, significant, net-positive effect under our experimental conditions with increased bath temperature. There are many factors involved in the sonoporation process which have temperature-dependent qualities which, together, may affect its efficacy. For instance, the cavitation of microbubbles is an important factor. Microbubble properties such as size distribution and stability have been shown to be affected by temperature [31, 32]. In [32], the mean microbubble diameter for SonoVue UCA (phospholipid-shell) was seen to increase with temperature and, after a few minutes, dropped abruptly. The growth of the microbubbles was thought to be occurring due to the gas expansion of the bubbles as well as the reduced surface tension of the shell. The phase transition temperature of the shell of SonoVue UCA has been noted to be around 40 °C [31]. The decreasing stability with time was attributed to the possibility of a phase change of the shell since a similar drop was not observed at 37 °C. Though the UCA used here (Definity) is not identical, there may be similarities in its temperature-dependent behavior. That is, the higher temperatures may have affected both the microbubble size and stability of the Definity UCA during treatment. Here, the total treatment time was 7 min whereas the abrupt drop in SonoVue population observed in [32] occurred around 6 min. It may be the case that during the sequential insonations that the properties of the microbubbles changed between the exposures, affecting the average per-replicate transfection efficiency and cell detachment at the elevated temperatures.

Temperature-dependent qualities of the cell membrane such as membrane fluidity have been seen to affect sonoporation efficacy as well. In [33], prostate cancer-derived PC-3 cells treated with a heat treatment (44 °C, 1-min equalization) showed a 15-fold increase in the rate of transfection from sonoporation treatment over 37 °C. This is in contrast to the effect observed here. The authors attributed the increase in transfection to an increase in membrane fluidity as the effect was similar to lidocaine treatment, a substance known to increase membrane fluidity. The effect of temporal microbubble size and stability in [33] was likely minimal due to the short per-replicate insonations (1 min).

The magnitude and homogeneity of the acoustic field may have been affected at the fixed treatment distance due to a change in the speed of sound of the water bath at higher temperatures. Due to the device’s operation in the near field region, this effect would likely be more pronounced than if the treatment distances were closer to the near-far field transition distance (∼45 mm in Fig. 2). However, the change in speed of sound due to temperature from 37 to 42 °C is a relatively small change, approximately 0.53 % (1524 m/s v. 1532 m/s [34]).

In order to come to a conclusion on the effect of temperature on sonoporation efficacy on SiHa cells, these many factors must be subject to future study.

The sonoporation device, as it is presented here, only addresses a subset of the issues which impede the routine use of our previous system. The driving of the transducers still requires generalized electrical equipment such as an arbitrary waveform generator and an amplifier as well as manual switching and setup of these devices. Also, the current version lacks of a cavitation detector that can be used to better correlate bubble activity and transfection effects. Future version of the device will incorporate a small factor 6-channel amplifier and a cavitation detector, which can be easily incorporated in the current design. Nevertheless, the device addresses two important aspects of the previous system’s setup. First, the device currently eliminates the time and training required for setting up the acoustic conditions for the experiment. The fixed positions of the transducers need to be set once during construction, after characterization. Once the transducers are in a fixed position, the acoustic conditions are maintained between experiment days without intervention by the operator. Second, the device’s compact size reduces the requirement for water bath size and, in turn, reduces the filling, degassing, heating, and emptying times required. These two aspects of the previous system likely have the most significant impact on the time and training required to set up an experiment.

Automating and providing a convenient user interface for the driving of the six transducers has yet to be solved and is the subject of future work. Doing so would allow for the six transducers to expose the six target areas of the Opticell in a concurrent, interlaced, or sequential manner as well as allow for per-transducer driving conditions. Concurrent exposure is potentially possible given the directivity of the transducers (Fig. 4). This scheme would reduce further the total time required to perform an experiment by reducing the time to perform sonication from 7 to 1.5 min per Opticell as well as avoid differences in the conditions between exposures due to temporal changes.

There are commercial systems as well which aim to make performing sonoporation studies easier. For example, the Sonidel SP100 (Sonidel Ltd., Dublin, Ireland) is a sonoporator which is commercially available. This device consists of a free ultrasound transducer, driving electronics, and easy-to-use interface. While this device been used in a number of sonoporation studies, it often appears to be used with “modification” [17, 19]. The acoustic conditions at the cells is not necessarily ensured in its stock configuration due to the use of a hand-held ultrasound transducer. That is, this sonoporator still requires an additional experimental apparatus to ensure consistent ultrasound exposure between experiments. Hence, this system fails to address the same issues as the device presented here.

Unfortunately, Opticells have recently been discontinued. However, the concept of a device for performing sonoporation on adherent cell cultures which minimizes operator expertise could certainly be adapted for a suitable alternative cell culture system if one were to become available.

## Conclusions

The design of a prototype device to minimize the time and expertise required to perform in vitro sonoporation on monolayer cell cultures was devised, characterized, and experimentally validated. A preliminary study of sonoporation of SiHa cells under mild hyperthermic conditions did not show a significant effect on transfection efficiency or viability over normal conditions. The prototype device has been shown to be effective for use in sonoporation studies and will provide a reduction in the time and training required to perform these types of studies in the future.

## Abbreviations

US: ultrasound
UCA: ultrasound contrast agent
HPV: human papillomavirus
DNA: deoxyribonucleic acid
RNA: ribonucleic acid
pDNA: plasmid DNA
GFP: green fluorescent protein
PARP: poly ADP-ribose polymerase
DAPI: 4’,6-diamidino-2-phenylindole
